# *Actinobaculum massiliense* Proteome Profiled in Polymicrobial Urethral Catheter Biofilms

**DOI:** 10.3390/proteomes6040052

**Published:** 2018-12-09

**Authors:** Yanbao Yu, Tamara Tsitrin, Harinder Singh, Sebastian N. Doerfert, Maria V. Sizova, Slava S. Epstein, Rembert Pieper

**Affiliations:** 1 J. Craig Venter Institute, 9605 Medical Center Drive, Rockville, MD 20850, USA; yayu@jcvi.org (Y.Y.); tvtsitrin@yahoo.com (T.T.); hsingh@jcvi.org (H.S.); 2 College of Science, Northeastern University, 360 Huntington Avenue, Boston, MA 02115, USA; doerfert@gmail.com (S.N.D.); maria.v.sizova@gmail.com (M.V.S.); slava.epstein@gmail.com (S.S.E.)

**Keywords:** Keywords: actinobaculum, urinary tract infection, biofilm, catheter, CAUTI, host-pathogen interaction, proteome, polymicrobial, metabolism, uropathogen

## Abstract

*Actinobaculum massiliense*, a Gram-positive anaerobic coccoid rod colonizing the human urinary tract, belongs to the taxonomic class of Actinobacteria. We identified *A. massiliense* as a cohabitant of urethral catheter biofilms (CB). The CBs also harbored more common uropathogens, such as *Proteus mirabilis* and *Aerococcus urinae*, supporting the notion that *A. massiliense* is adapted to a life style in polymicrobial biofilms. We isolated a clinical strain from a blood agar colony and used 16S rRNA gene sequencing and shotgun proteomics to confirm its identity as *A. massiliense*. We characterized this species by quantitatively comparing the bacterial proteome derived from in vitro growth with that of four clinical samples. The functional relevance of proteins with emphasis on nutrient import and the response to hostile host conditions, showing evidence of neutrophil infiltration, was analyzed. Two putative subtilisin-like proteases and a heme/oligopeptide transporter were abundant in vivo and are likely important for survival and fitness in the biofilm. Proteins facilitating uptake of xylose/glucuronate and oligopeptides, also highly expressed in vivo, may feed metabolites into mixed acid fermentation and peptidolysis pathways, respectively, to generate energy. A polyketide synthase predicted to generate a secondary metabolite that interacts with either the human host or co-colonizing microbes was also identified. The product of the PKS enzyme may contribute to *A. massiliense* fitness and persistence in the CBs.

## 1. Introduction

*Actinobaculum massiliense* is a Gram-positive, facultatively anaerobic coccoid rod and apparently rare pathogen able to infect the human urinary tract [1]. A case report described the species as the cause of catheter-associated recurrent cystitis in an elderly female patient with resistance to the antibiotics trimethoprim/sulfamethoxazole and rifamycin while sensitive to doxycycline [1]. A different report associated *A. massiliense* with urosepsis [2]. The bacterium is taxonomically part of the order Actinomycetales and class Actinobacteria. Twenty years ago, the genus *Actinobaculum* was distinguished from Actinomycetes and Arcanobacteria by Lawson et al. based on 16S rRNA gene sequence comparisons [3]. Evaluating the literature, the most common opportunistic pathogen of this genus is *Actinobaculum schaalii* which has been associated with urinary tract infection (UTI), catheter-associated UTI (CAUTI), abscesses, urosepsis, and bacteremia [4,5]. Due to the fastidious growth under aerobic conditions and morphological similarity to commensal urogenital organisms, *A. schaalii* may be a more frequent cause of UTI, asymptomatic bacteriuria (ASB) and CAUTI than current epidemiological data suggest [4]. By interpreting 16S rRNA and DNA hybridization data, *Actinobaculum spp.* were reclassified as *Actinotignum spp.,* including *Actinotignum schaalii* [6]. Data for *A. massiliense* strains, deposited as strains CCUG 47753(T) and DSM 19118(T), suggest that some strains belong to the species *A. schaalii* while others represent a new species termed *Actinotignum sanguinis* [6]. Given the uncertainty of *A. massiliense* strain assignments to a genus, we use the original taxon, *Actinobaculum massiliense*, in the context of this report. In addition to 16S rRNA analyses, mass spectrometry-based microbial identification methods such as MALDI-TOF were introduced resulting in more frequent identifications of *Actinotignum/Actinobaculum spp.* from clinical urine isolates [7].

The growth of *Actinotignum/Actinobaculum spp.* in an aerobic milieu on blood agar in vitro has been successful occasionally. Anaerobic culture appears to result in improved recovery rates in the form of small, gray colonies on blood agar plates [5]. The complete genome sequence of the *A. schaalii* strain CCUG 27420 was published in 2014 [8]. Draft genome sequences of *A. massiliae* (*massiliense*) - strains FC3 [9] and ACS-171-V-COL2, submitted to the EMBL/GenBank/DDBJ databases in 2012 [10] - were reported. Reference proteomes with 1444 protein sequences (*A. schaalii*; no. UP000035032) and 1696 sequences (*A. massiliense*; no. UP000009888) were deposited in the UniProt proteome database. Genome analyses of *A. schaalii* CCUG 27420 and *A. massiliense* FC3 revealed genes for fimbriae and capsule formation [8] and for bacteriocin and toxin-antitoxin systems [9], respectively. The data suggest that *A. massiliense* has acquired more genes via lateral gene transfer than *A. schaalii*, while the species also share several putative virulence factors [9]. While clinical strains were linked to several CAUTI cases, the persistence of *Actinotignum/Actinobaculum spp.* on the catheter surface as biofilm residents has not been studied to date. To our knowledge, little is known about their metabolic adaptations to the human urinary tract and urine nutrient resources. Metabolism and fitness changes of bacteria in the urinary milieu, with most data derived from uropathogenic *E. coli*, were reviewed [11]. Major adaptations are siderophore production, due to bivalent metal ion sequestration by the human host, and high expression levels of transport systems and metabolic pathways for metal ions, arginine and branched-chain amino acids, di- and oligopeptides, and mannonate and galactoside sugars. Sugar isomerization in the pentose phosphate pathway was also reported to be important for *E. coli* fitness in the urinary tract [11]. Sialic acid availability in urine (in soluble and mucosal surface glycoproteins) is controlled by enzymes such as NanA and influences phase switching of *E. coli* fimbriae and mucosal adhesion [11]. Differences in the requirement of glycolysis were reported for *E. coli* vs *P. mirabilis*. Mutations in the glycolytic pathway of *P. mirabilis* resulted in in vivo fitness defects in an ascending UTI model [12]. Cultivation of *E. faecalis* in urine revealed high abundances of potential adhesins and peptide transporters, suggesting adaptations in transport and cell surface properties [13].

The goal of this investigation was to characterize *A. massiliense* isolates residing in urethral polymicrobial CBs with respect to energy metabolism, expression of potential virulence and fitness factors and the microbial-host immune cell crosstalk in the biofilm milieu. We integrated proteomics and bioinformatics approaches to gain the first insights into how this clinically rarely identified bacterium interacts with other bacteria and the human host.

## 2. Methods

### 2.1. Ethics Statement

A human subject protocol, together with a consent form explaining risks of participation in the study, was generated by the Southwest Regional Wound Care Center (SRWCC) in Lubbock, TX, USA and the J. Craig Venter Institute (JCVI) in Rockville, MD, USA. The study number was #56-RW-022. The Western Institutional Review Board (WIRB) in Olympia, Washington and the IRBs of the JCVI and Northeastern University (NEU) in Boston, MA, USA approved the protocol in 2013. All enrolled adults provided written consent. Catheter specimens were collected firsthand for the study. A medical need to replace the Foley catheters in patients in the context of bladder management existed. Scientists who analyzed specimens via microbial culture and proteomic analyses (at the research sites JCVI and NEU) did not have access to data allowing patient identification. Electronic and printed medical records created at SRWCC were retained for four years to facilitate integrated reviews of medical data and scientific research results and then destroyed.

### 2.2. Clinical Background and Patient Specimens

The parent study was prospective and included nine patients who contributed indwelling catheter samples collected longitudinally over a time frame of 3 to 6 months. The patients suffered from neurogenic bladder syndrome as well as chronic wounds. Treatment of these pathologies were the reason for regularly scheduled visits of the wound clinic. Routine care included catheter exchange to minimize the risk of CAUTI. By performing 16S rRNA and proteomic analyses of series of catheter extracts, the genus *Actinobaculum* was identified from CB samples derived from two male subjects (P1 and P5). The Foley catheter material was latex in each case.

### 2.3. Catheter Sample Processing for Microbial Cultures, 16S rRNA Sequencing, and Proteomics

Catheter specimens were cut into one-inch pieces. Two methods were used to process catheter samples, one with the objective to proceed with culture-free metagenomic and proteomic analyses and the other with the objective to isolate and grow fastidious anaerobic and microaerophilic microbes. Immediately after collecting the catheter specimens, samples for culture-free ‘omics analyses were placed in polypropylene tubes, stored at –20 °C for 6–24 h prior to shipment to JCVI on ice and frozen at −80 °C until the day of sample extraction. For the culture of fastidious microbial organisms, samples were preserved differently. Freshly collected catheter specimens were placed into Balch glass test tubes filled with 10 mL sterile anaerobic basic medium with urea (BMU). The tubes were flushed with nitrogen gas, sealed with a rubber stopper, capped, and delivered to the lab at NEU via overnight shipment at ambient temperature. BMU media (pH 6) contained the following components in g/L: yeast extract, 0.1; Casamino Acids, 0.1; KH_2_PO_4_, 2.1; K_2_HPO_4_, 6.35; urea, 0.1; MgCl_2_ × 6 H_2_O, 0.1; NH_4_Cl, 0.4; CaCl_2_ × 2 H_2_O, 0.05; trace elements SL10, 1 ml/L; FeCl_2_ × 4 H_2_O, 0.05; l-cysteine-HCl, 0.5; resazurin 0.0025. Upon arrival of the tube in the lab, it was placed in an anaerobic glove cabinet, vortexed to homogenize the microbial suspension and serially diluted. BMU dilutions were plated onto anaerobic trypticase-yeast extract agar plates supplemented with L-cysteine-HCl as a reducing agent and sheep blood (25 mL/L). The trypticase-yeast extract (TY) agar composition in g/L was as follows: trypticase, 20.0; yeast extract, 10.0; agar, 15; FeCl_2_ × 4 H_2_O, 0.05; l-cysteine-HCl, 0.5. To propagate growth of fastidious anaerobic bacteria, fresh samples were also inoculated into BMU liquid media supplemented with 1% of human serum, incubated at 37 °C for 7–10 days and then plated on anaerobic TY-blood agar.

### 2.4. In Vitro Liquid Culture of Actinotignum massiliense in Rich Growth Media

After up to 10 days of incubation, single colonies were picked from plates with a sterile loop and re-inoculated into liquid TY media supplemented with 1% of human serum for sub-culturing. All steps were conducted in an anaerobic glove box. From sub-cultures of one facultative anaerobic bacterial colony, *A. massiliense* was identified by 16S rRNA sequencing. From a culture stock (a TY agar colony stored in trypticase-yeast extract at −80 °C), the bacterium was grown anaerobically in 10 mL liquid trypticase soy broth (#43592; Sigma-Aldrich) without agitation overnight at 37 °C. The cell density (OD_600_) of the culture was not determined. The cells were collected via centrifugation at 3200× *g* for 15 minutes at ambient temperature and washed with PBS prior to storage of the cell culture pellet (CCP) at −80 °C and shipment to JCVI.

### 2.5. Catheter Biofilm Extraction

Urethral latex catheter pieces were thawed. Additional urine pellet (UP) samples derived from catheter bags that the patients used were also available and processed as reported previously [14]. Each catheter piece was thawed and placed in a 15 mL Falcon tube with 2–3 mL CHO buffer (100 mM sodium acetate, 20 mM sodium meta-periodate and 300 mM NaCl; pH 5.5). At ambient temperature, the CHO-suspended catheter piece was sonicated in a water bath for 10 minutes allowing the CB biomass to detach from the latex surface and subsequently vortexed. Residual biomass was scraped off the surface with a plastic spatula. The sonication and vortex steps were repeated. The pH of this extract was adjusted to ~6.5 to 7.5 with 1 M Tris-HCl (pH 8.1). Centrifugation at 8000× *g* for 15 minutes allowed the recovery of a supernatant (CB_sup_) and a pellet (CB_pel_) fraction. The volume of the CB_sup_ fraction was reduced to ~ 0.5 ml in an Ultrafree-4 filter unit (10 kDa MWCO) via centrifugation at 3200× *g* followed by a buffer exchange into PBS. The CB_pel_ fraction was not re-suspended. Both fractions were stored at −80 °C until further use.

### 2.6. Cell Lysis and Preparation of CCP and CB Lysates for Proteomics

CB_pel_, UP and CCP samples were lysed with SED solution (1% aqueous SDS, 5 mM EDTA and 50 mM DTT) in low-protein binding microcentrifuge tubes in a 1:5 volume ratio. Each sample was sonicated in a Misonex 3000 water bath sonicator (ten 30 s on/off cycles at amplitude 6.5), then moved to a heat block (95 °C) for three minutes, and finally incubated to complete lysis with intermittent vortex steps at 20 °C for 15 minutes. Lysates were cleared by centrifugation at 13,100× *g* for 10 minutes. Aliquots were subjected to SDS-PAGE to visualize protein bands and estimate the total protein concentration by staining with Coomassie Brilliant Blue-G250 (CBB). A 2 µg BSA lane served as a standard to estimate the protein quantity from the CBB-stained lane intensity for a lysate. CB_sup_ fractions were also run in SDS-PAGE gels. Lysate and CB_sup_ aliquots containing approximately 100 µg total protein were subjected to filter-aided sample preparation (FASP) in single-tube Vivacon filters (10 kDa MWCO membrane; Sartorius AG, city, Germany), and sequencing-grade trypsin was used to degrade the proteins as reported previously [15]. FASP-processed peptide mixtures were desalted using the Stage-Tip method [16]. The peptide mixtures were lyophilized and then ready for LC-MS/MS analysis.

### 2.7. Shotgun Proteomics Using LC-MS/MS

Lyophilized peptide mixtures were re-suspended in 10 μl 0.1% formic acid (solvent A). The LC-MS/MS workstation was composed of the LTQ-Velos Pro ion-trap mass spectrometer coupled to the Easy-nLC II system via a FLEX nano-electrospray ion source (Thermo Scientific, San Jose, CA, USA). Detailed LC-MS/MS analysis steps were previously described [17]. The sample was loaded onto a C_18_ trap column (100 μm × 2 cm, 5 μm pore size, 120 Å) and separated on a IntegraFrit^TM^ C_18_ analytical column (75 μm × 15 cm, 3 μm pore size, 150 Å (New Objective, Woburn, MA, USA) at a flow rate of 200 nL/min. Starting with solvent A, a linear gradient from 10% to 30% solvent B (0.1% formic acid in acetonitrile) over 195 minutes was followed by a linear gradient from 30% to 80% solvent B over 20 minutes and re-equilibration with solvent A for five minutes. The column was washed thrice with a 30-minute solvent A to B linear gradient to minimize carry-over of peptides from sample to sample. Peptide ions were analyzed in a MS^1^ data-dependent mode to select ions for MS^2^ scans using the software application XCalibur *v2.2* (Thermo Scientific). The ion fragmentation mode was collision-activated dissociation with a normalized collision energy of 35%. Dynamic exclusion was enabled. MS^2^ ion scans for the same MS^1^ *m*/*z* value were repeated once and then excluded from further analysis for 30s. Survey (MS^1^) scans ranged from the *m*/*z* range of 380 to 1800 followed by MS^2^ scans for the selected precursor ions. The ten most intense peptide ions were fragmented in each cycle. Ions that were unassigned or had a charge of +1 were rejected from further analyses. At least two technical LC-MS/MS replicates were run for a sample. Raw MS files from the replicate analyses were combined for the database searches.

### 2.8. Computational Methods to Profile and Quantify the Metaproteomes

Raw MS files were searched using the Sequest HT algorithm integrated in the software tool Proteome Discoverer v1.4 (Thermo Scientific). Technical parameters and database construction have been described previously [18,19]. Only rank-1 peptides with a length of at least seven amino acids were considered for analysis. The FDR rates were estimated using the Percolator tool in Proteome Discoverer v1.4 with a (reverse sequence) decoy database. Protein hits identified with a 1% FDR threshold were accepted, and the ‘protein grouping’ function was enabled to ensure that only one protein was reported when multiple proteins shared the same set of identified peptides. The initial database searches without prior knowledge of the present microbial organisms were performed using reviewed protein entries of the non-redundant Homo sapiens UniProt dataset (release 2015-06; 20,195 sequences, www.uniprot.org) and sequence entries for 23 microbial genomes, available from the UniProt proteome data repository, for common urogenital tract-colonizing microbial species including *A. schaalii* strain CCUG 27420 (UniProt ID UP000035032) (Supplemental File S1). Based on the initial results, datasets with more than ten identified *A. schaalii* proteins were re-analyzed with modifications to the database to verify the *Actinotignum/Actinobaculum spp.* identity in the samples. Two additional species were reported to colonize the human host: *A. urinale* (strain UMB0759; UniProt ID UP000235308) *and A. massiliense* (strain ACS-171-V-COL2; UniProt ID UP000009888). A third stage of computational analysis pertained to using only those species which, based on 16S rRNA and preliminary proteomic results, were confidently identified in a sample. This multi-step approach served to minimize incorrect assignments of identical peptides to proteins of origin by the Proteome Discoverer software. This is important when orthologous proteins with high sequence identities are present in a database, typically in the context of phylogenetically highly related organisms. For the quantitative proteomic analysis, CB_sup_ and CB_pel_ Proteome Discoverer v1.4 output files were merged. For the purpose of this study, we selected the peptide-spectral match (PSM) counts pertaining to the *A. massiliense* proteome to compare clinical (CB) and cell culture (CCP) datasets. Human proteins in the CB datasets were also quantified. The normalization of protein quantities across all samples was done based on the division of the PSM for a protein (protein i) by the sum of all PSMs for a given species (*A. massiliense* or human) in that dataset (PSMi/∑PSM).

### 2.9. 16S rRNA Analysis

All CB_pel_ samples (5–25 µL volume) were re-suspended in 300 µL TES buffer (20 mM Tris-HCl, pH 8.0, 2 mM EDTA, and 1.2% Triton X-100), vortexed occasionally and incubated at 75 °C for 10 minutes. To the cooled CB_pel_ suspension, 60 µL chicken egg lysozyme (200 µg/mL), 5.5 µL mutanolysin (20 U/mL; MilliporeSigma, Billerica, MA, USA), and 5 µL linker RNase A were added. An incubation for 60 min at 37 °C was followed by addition of 100 µL 10% SDS and 42 µL proteinase K (20 mg/mL). Bacteria in these suspensions were lysed overnight at 55 °C. A standard DNA extraction procedure with phenol-chloroform-isoamylalcohol (25:24:1), centrifugation at 13,100× *g* for 20 minutes and recovery of the aqueous phase to enrich bacterial DNA followed. Nucleic acids were salted out by adding 3 M sodium acetate (pH 5.2). DNA was precipitated by adding an equal volume of ice-cold isopropanol, pelleted by spinning at 13,100 × *g* for 10 minutes, washed with 80% ethanol, and resuspended in TE buffer for storage at –20 °C. DNA library preparation for the amplification of V1-V3 regions of 16S rRNA bacterial genes and the MiSeq (Illumina, San Diego, CA, USA) sequencing approach were described previously [20]. With UPARSE for phylogenetic analysis [21], operational taxonomic units (OTUs) were generated de novo from raw sequence reads using the default parameters in UPARSE software, the Wang classifier and bootstrapping using 100 iterations. Taxonomies were assigned to the OTUs with Mothur applying the SILVA 16S rRNA database version 123 (www.arb-silva.de) as the reference database [22]. Unbiased, metadata-independent filtering was used at each level of the taxonomy by eliminating samples with less than 2000 reads.

### 2.10. Protein Function and Biological Pathway Analyses

The annotation of protein-encoding genes in the reference proteome *A. massiliense* ACS-171-V-COL2 [10] is preliminary, and many proteins are annotated as uncharacterized. We conducted sequence homology searches with BlastP (www.uniprot.org) to identify bacterial orthologs with gene identifiers and putative functional roles. The Metacyc.org and Ecocyc.org databases were used to infer relationships of proteins with biological pathways. Assessing both frequency of identification of proteins part of a pathway and relative abundance of the *A. massiliense* proteins, we inferred the overall activity of biosynthetic and metabolic pathways in the CB milieu. Assessing the predicted presence of signal sequences, cell wall localization motifs and transmembrane domains, as well as localization of genes in clusters, we obtained additional data indicative of a protein’s functional role.

## 3. Results

**Figure 1:**
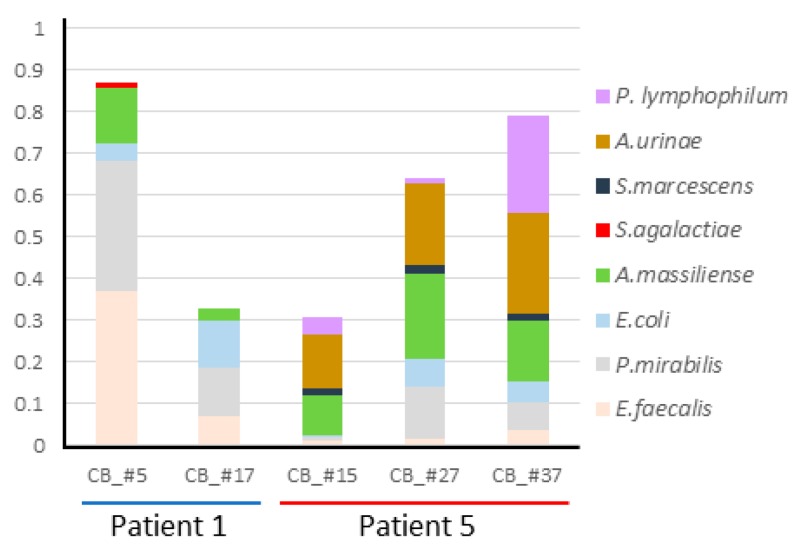
Quantitative representation of microbial proteomes in CB samples. The bars are ordered from left to right according to the sequential collection time points. For patient 1, the time points were a week apart; for patient 5, the time points were a month apart. Number gaps do not indicate missed samples. Colored segments of bars represent the relative contribution of a microbial species to the entire proteome. The panel of bacterial species on the right provides the color code for the species as shown in the segmented bar diagram.

*Actinobaculum massiliense* recurrently colonizes urethral catheter surfaces. For patients P1 and P5 who were part of a study to understand the microbial complexity of urethral CBs in a hostile host milieu, we observed *A. massiliense* to be a cohabitant of polymicrobial biofilms. In three sequentially replaced catheters from P1, *A. massiliense* was a component of the CB community joined by *Proteus mirabilis*, *Escherichia coli*, and *Enterococcus faecalis*. In five sequentially replaced catheters from P5, the bacterium colonized CB surfaces together with all or some of the following species: *P. mirabilis*, *E. coli*, *E. faecalis*, *Aerococcus urinae*, *Streptococcus agalactiae*, and *Propionimicrobium lymphophilum* (Figure 1). 16S rDNA surveys are sensitive with respect to amplification of the *Actinotignum/Actinobaculum* 16S rRNA V1–V3 region. This data indicated presence of this genus in six and eight sequentially collected CB and UP samples from patients P1 and P5, respectively, confirming the proteomic results. Re-identification of several species in sequential catheters supports the notion that *A. massiliense* and its cohabitants colonized the indwelling urethral catheters recurrently, in contrast to an alternative outcome: the colonization by new strains ascending from the urethral meatus that happen to be from the same species. This data is indicative of the stability of long-term polymicrobial communities, reminiscent of reports on *P. mirabilis*-dominated crystalline biofilms [23]. The urine pH values were close to neutral. The *P. mirabilis* strains in P1 and P5 did not express urease, the enzyme converting urea into ammonia and alkalinizing the urine pH of urine, at detectable levels. The patients were not systemically treated with antibiotic drugs during the time of specimen collection according to the medical reports. We infer that the bacterial biofilm persisted via adhesion to the urethral mucosa during the patients’ catheter replacements and moderately altered their quantitative compositions over time (Figure 1). Both patients did not report symptoms, which is common in neurogenic bladder syndrome cases. The clinical diagnosis was catheter-associated asymptomatic bacteriuria. Data from matching UP specimens for each timepoint (not shown in Figure 1) revealed lower microbial contents relative to the total proteomes for all taxa, supporting the notion that *A. massiliense* was a biofilm cohabitant rather than adventitiously present due to planktonic growth in urine. Actinobacteria (*A. massiliense* and *P. lymphophilum* in P5) were variably abundant in catheter extracts, revealing a pattern of dynamic co-habitation with other bacterial species and potential metabolic co-dependencies.

**Figure 2:**
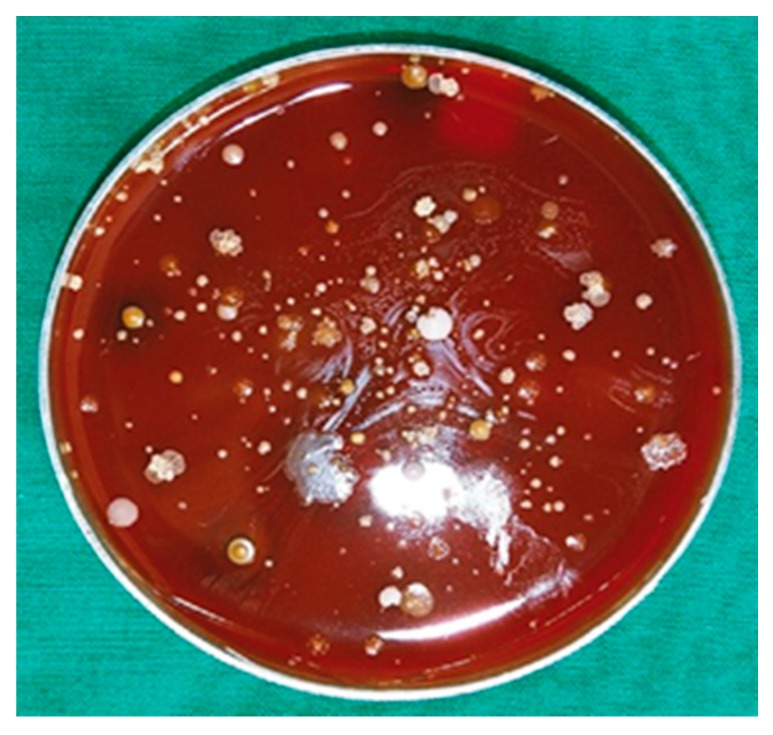
Anaerobically grown microorganisms derived from a urethral catheter sample of patient 5 on a blood agar plate. Within 48 hours of growth, various colonies emerged. Among those identified by 16S rRNA analysis on the genus or species level were: *Actinobaculum massiliense*, *Actinomyces sp., Aerococcus sp.*, *Enterococcus sp.*, *Escherichia coli*, *Finegoldia sp., Morganella morganii*, *Porphyromonas asaccharolytica*, and *Prevotella timonensis*.

*Actinobaculum massiliense* is a fastidious bacterium that favors anaerobic growth. A few of the clinical specimens were subjected to microbial culture on blood agar preserving and growing the viable microbes under anaerobic or aerobic conditions. We did not isolate *A. massiliense* colonies from catheter specimens frozen and re-thawed, followed by incubation under ambient air conditions or with 5% CO_2_. We isolated *A. massiliense* colonies after preserving the catheters in nitrogen-flushed and sealed tubes to maintain anaerobicity followed by growth within 24 h on blood agar in an anaerobic chamber. Small grey colonies became visible on blood agar and were isolated after 48 h of growth and analyzed by 16S rRNA sequencing. Some of the colonies were identified as *A. massiliense*. As the image of Figure 2 shows, many bacterial species with distinct colony morphologies grew in the anaerobic milieu, consistent with the low levels of oxygen that the urethral catheter biofilm-colonizing microbes are exposed to, on blood agar.

Proteomic analysis of *A. massiliense* from the growth in nutrient-rich media and clinical samples. To our knowledge, we characterize the proteome of any *Actinotignum/Actinobaculum* species, derived from in vitro or in vivo growth environments, for the first time. We examined four *A. massiliense* proteomic datasets associated with CB extracts, two each pertaining to samples from patients P1 and P5. Their composition was expected to reflect bacterial adaptation to the nutrient conditions in the urinary tract. In addition to inorganic salts, urine is rich in urea, organic and amino acids. This body fluid contains peptide breakdown products of proteins, glucuronate-conjugated toxins and pigments (e.g., urobilin) that are mostly derived from renal metabolic and excretion processes [24]. Urothelial mucosal surface glycoproteins and breakdown products and the CB matrix also contribute to the pool of nutrients. We compared the in vivo proteome with an isolate of *A. massiliense* (P5) that was grown planktonically to stationary phase in sugar- and amino acid-rich growth media. The analyses do not discern which proteomic and, by inference, metabolic changes are attributable to exposure to urine as a nutrient versus the microbial cellular and extracellular matrix environment within the biofilm. All proteomic datasets are provided in the Supplemental File S2, with the identities of proteins listed as annotated in the genome of strain ACS-171-V-Col2 and their PSM-based quantities. MS raw files are deposited in the EMBL-EBI archive PRIDE via ProteomeXchange (www.ebi.ac.uk/pride/archive) with the identifier PXD011327. Overall, 759 proteins with at least two unique peptides, representing 44.7% of the in silico predicted proteome, were identified; 739 and 585 proteins were identified from the in vitro and the combined in vivo datasets, respectively. In vivo vs. in vitro abundance differences were observed for many proteins, in support of the notion that *A. massiliense* modulates its proteome to thrive as a constituent of a biofilm. It competes with other bacteria for nutrients in a host milieu that features prolonged infiltration of innate immune cells in the urothelial mucosa. Due to limited protein functional descriptions for 1696 unreviewed TremBL entries (www.uniprot.org) predicted for the genome of strain ACS-171-V-Col2, we assessed the biological roles of proteins based on the presence of conserved domains (information in UniProt) and sequence homology searches to identify better annotated orthologs. Furthermore, we inferred the subcellular localization of some proteins from transmembrane, secretion signal and cell wall anchor motifs. We prioritized proteins that potentially interact with the human host and/or are important for bacterial fitness in the urinary system. The list of these proteins, many of which were found to be abundant in vivo, is contained in Table 1.

**Figure 3:**
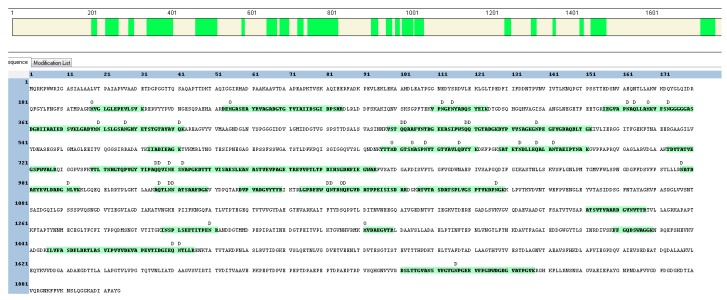
HMPREF9233_01095 protein sequence (a putative subtilisin-like protease). The protein segments from the N- to C-terminus and the peptides identified by shotgun proteomic analysis are highlighted in green (in the bar at the top of the graphic and in amino acid sequence format below, respectively). Modifications are listed above the amino acid position, including deamidation (D) and methionine oxidation (O). These modifications may have occurred during sample processing steps and not reflect biological changes.

Potentially direct *A. massiliense* protein interactions with the host environment. Two putative subtilisin-like proteases (gene loci 01095 and 00810; gene locus prefixes provided in Table 1 footnote) were abundant in CB (in vivo) samples. The protease 01095 was on average 230-fold more abundant in vivo than in cell culture (in vitro), the protease 00810 was 2.4-fold more abundant in vivo. Figure 3 shows the sequence coverage for the protease 01095 from a P5 dataset. Lack of sequence coverage in the 200 *N*-terminal amino acids suggests a pre-proenzyme, with a signal peptide cleavage site at A_29_D and a propeptide ranging from D_30_ to ~K_197_ that is cleaved to enable enzyme activation. The two proteases have 32% sequence identity (BlastP E-values of 1 × 10^–97^ and 8 × 10^–104^, respectively) with lactocepin, a *Lactobacillus paracasei* protease that exerts anti-inflammatory activitiy by degrading proinflammatory cytokines in the human gastrointestinal tract [25]. Of note, our proteomic data show evidence of complement system activation and infiltration of neutrophils in the urinary tract of P1 and P5. Effector proteins and cytokines released by innate immune cells into the urinary tract may be susceptible to cleavage by these subtilisin-like proteases [26]. Table 2 lists the twenty-five on average most abundant human proteins in the four in vivo datasets. 

**Figure 4:**
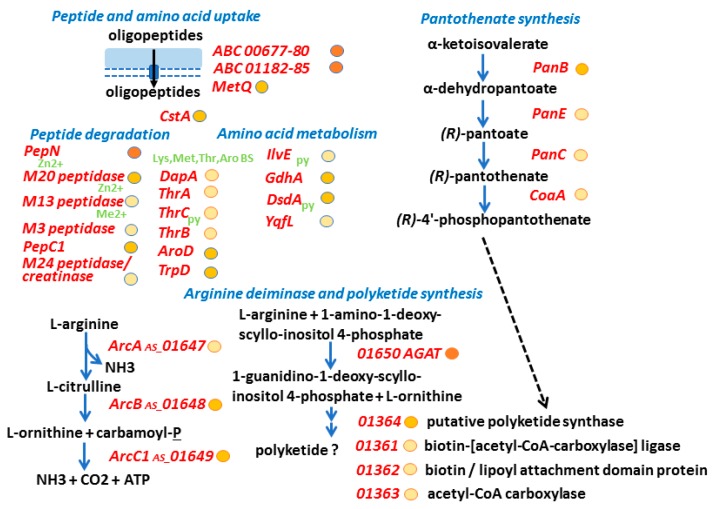
Putative *A. massiliense* peptide uptake, peptide/amino acid metabolism and PKS synthesis functions. The schematic contains protein names in red (short names as annotated in strain ACS-171-V-Col2 database or for orthologs) and/or gene loci (gene locus prefix HMPREF9233_ is not added to the five-digit accession number). The metabolite names are given in black, blue arrows show an enzymatic activity, black arrows a transport activity, and hatched black arrows a cofactor contribution to an enzyme. We provide approximate abundance values of the in vivo detected proteins using circles (behind their names). The darker the fill color, the higher the average abundance level of a protein in averaged in vivo datasets. Proteins predicted to contain cofactors (based on evidence from characterized orthologs) have green symbols underneath/behind the protein names: Me^2+^ (metal ion), Zn^2+^ (zinc), py (pyridoxal-5’-phosphate). Other acronyms: ABC, ABC transporter; Lys, Met, Thr, Aro (aromatic acid) BS, enzymes involved in the biosynthesis of amino acids.

Two Rib/alpha/Esp surface antigen repeat-containing proteins encoded by adjacent gene loci (00826 and 00827) were also abundant in CB proteomes. The protein 00826 was on average 13-fold more abundant in vivo than in vitro, the protein 00827 was 3.8-fold more abundant. Both have Ca^2+^-dependent cadherin-like domains known to promote cell adhesion, and are truncated compared to a better characterized ortholog, the *E. faecalis* Esp surface protein (EF0056). The sequences of Esp and 00826 are 39% identical in the aligned protein region (e-value 0.018). The *Enterococcus faecalis* Esp promotes formation of biofilms on catheter bag surfaces [30]. The protein 00826 has calcium-binding type 3 (T3) repeats. ORF 00828 (82 amino acids) has a LPHTG motif suggesting sequence/assembly errors. A gene with ORFs 00827 and 00828 would encode a single cell wall-anchored protein with an adhesion function. Two additional LPXTG motif proteins (gene loci 00581 and 00866) whose N-terminal domains have no functional role predictions were identified. In LPXTG peptide sequences, carboxyl termini of the threonine residue are coupled to amino groups of a peptide side chain part of the cell wall peptidoglycan. N-terminal segments of such proteins are typically exposed at the bacterial cell surface and interact with the extracellular environment. The protein 00581 was more abundant in in vivo proteomes compared to that of *A. massiliense* grown in vitro. A high M_r_, putative polyketide synthase (PKS), located in gene locus 01364, was expressed in vivo and in vitro. Interestingly, along with several enzymes participating in arginine metabolism, a L-arginine:glycine amidinotransferase was highly abundant in vivo. An ortholog of the enzyme was reported to contribute to the biosynthesis of a *Cylindrospermopsis raciborskii* polyketide hepatotoxin [31]. Enzymes expressed from two gene clusters and possibly involved in the biosynthesis of an *A. massiliense* polyketide are shown in the schematic of Figure 4. PKSs require a pantothenate cofactor for thiol transfer reactions, and enzymes part of a pathway to generate this cofactor were also expressed in vivo. Whether a functional link between the PKS and the amidinotransferase exists that results in the production of a uncharacterized polyketide secondary metabolite remains to be determined. Such a metabolite may act as a siderophore or cytotoxin, representing functions in metal ion acquisition and inter-microbial competition, respectively.

*A. massiliense* pathways with potential roles in nutrient acquisition in CB milieu. By surveying proteomic data of *A. massiliense* in clinical samples for putative transport functions, we identified the oligopeptide transporters encoded by the gene loci 00677–00680 and 01182–01185 (Figure 4). Of the two (most abundant) Mpp-type substrate binding proteins (00680 and 01185), 01185 was moderately increased in CBs compared to in vitro growth. The Mpp domain assignment suggests the import of oligopeptides or heme. Heme and metal ions are sequestered by the human host to prevent bacterial growth. In Table 2, two such proteins, LTF (which sequesters Fe^3+^) and S100-A8 (which sequesters Ca^2+^), are listed. A predicted periplasmic Fe/B12-binding protein (00649) was also identified (Table 1). It was detected in the proteome of biofilms from patient P5 but absent in biofilms from P1 and in vitro. If the Mpp-type ABC transporters indeed bind oligopeptides, *A. massiliense* would have not only the protein repertoire to import them but also to digest the peptides: the peptidases M13, M20, PepC1, and PepN and several enzymes degrading amino acids (GdhA, DsdA, and IlvE) were of moderate to high abundance in the in vivo datasets (Figure 4). PepC1, PepN, and M20 have zinc as a cofactor, indicating the need for metal ion uptake to support the metabolic processes.

**Figure 5:**
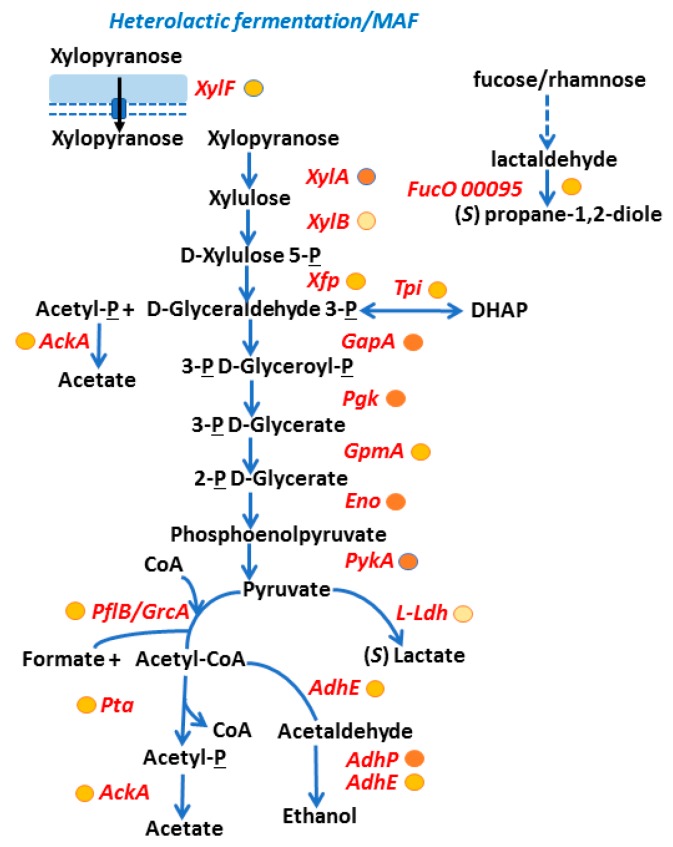
Evidence of *A. massiliense* mixed acid fermentation pathways utilizing xylose in vivo. The protein entities have acronyms/names as explained in the legend of Figure 4. The metabolite names are given in black, blue arrows show an enzymatic activity, black arrows a transport activity, and hatched blue arrows a multi-step metabolic pathway unresolved for this bacterial species. We provide approximate abundance values of in vivo detected proteins using circles (shown behind their names). The darker the fill color, the higher the average abundance level of a protein in in vivo datasets. Acronyms: MAF, mixed acid fermentation; DHAP, dihydroxyacetone phosphate; P, phosphate.

**Figure 6:**
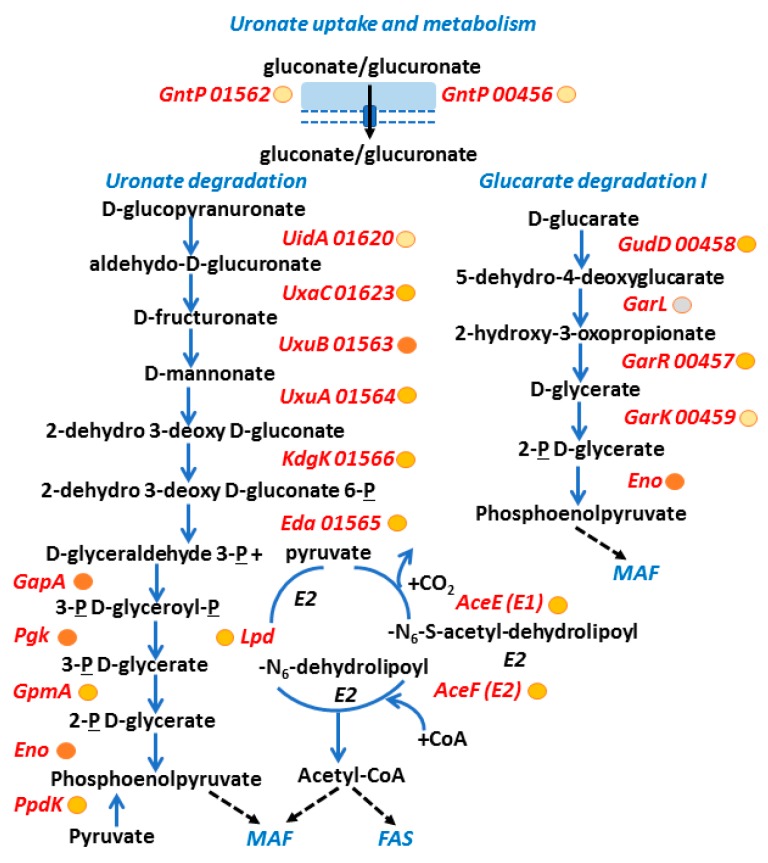
Evidence of active *A. massiliense* glucuronate and glucarate metabolism pathways in vivo. The protein entities have acronyms/names as explained in the legend of Figure 4. Metabolite names are given in black, blue arrows show an enzymatic activity, black arrows a transport activity, and hatched black arrows a link of a metabolite to a different catabolic pathway. We provide approximate abundance values of in vivo detected proteins using circles (behind their names). The darker the fill color, the higher the average abundance level in vivo. Grey-filled circle: protein not detected in the analyzed *A. massiliense* proteomes. Acronyms: MAF, mixed acid fermentation; FAS: fatty acid synthesis; P, phosphate; CoA, coenzyme A; E1/E2 cycle, pyruvate dehydrogenase complex.

*A. massiliense* glycolytic and mixed acid fermentation pathways. Among putative sugar uptake systems, the xylose uptake protein XylF, component of an ABC transporter, was highly expressed in CB proteomes. Further support for the importance of this transporter was derived from the fact that two enzymes, active in this sugar’s degradation pathway (XylA and XylB) and present in the same gene cluster (00910–00917), were also expressed in vivo. The aldolase Xfp which cleaves xylulose-phosphate was also abundant in the CB proteome. This pathway is shown in Figure 5. Downstream glycolytic and mixed acid fermentation (MAF) pathways, depicted in Figure 5, were highly active in in vivo proteomes and demonstrate the importance of the fermentation of sugars by *A. massiliense* in the low-oxygen environments in CBs. There was no evidence of an active bacterial citrate cycle. Fermentation activities also include the highly expressed enzyme FucO, which produces propan-1,2-diole (Figure 5). Furthermore, *A. massiliense* expressed proteins involved in the glucuronate/gluconate uptake and metabolic process. It also included a d-glucarate catabolic pathway. 2-Dehydro 3-deoxy d-gluconate appears to be fed into the glycolytic pathway via activities of KdgK and Eda, both of which were expressed in the CB milieu (Figure 6). Glucuronate conjugates are known to be produced in the human kidneys to detoxify and excrete hydrophobic waste products into urine. The pyruvate dehydrogenase complex, composed of AceE, AceF, and Lpd, also appeared to be active in vivo (Figure 6). Proteomic data yielded support for glycogen synthesis and glycogen mobilization by *A. massiliense* in vivo, as well as the synthesis of dTDP-L-rhamnose, a sugar present in many bacterial surface oligosaccharides (Supplemental File S3). The structures of rhamnose-containing cell surface antigens in *A. massiliense* are unknown. The bacterium also highly expressed glycolytic enzymes involved in the first steps of glucose and fructose catabolism in vivo (data not shown).

## 4. Discussion

*A. massiliense* persists in polymicrobial catheter biofilms in the presence of uropathogens. In five CB extracts derived from long term-catheterized patients whose urethral catheters were sequentially replaced, *A. massiliense* was identified as a cohabitant of polymicrobial CBs with ~5–30% microbial biomass contributions according to in vivo 16S rRNA and proteomics data. Whether *A. massiliense* is a true pathogen or bystander in cases of infection is a matter of debate [32]. Some case reports support the notion that the phylogenetically related *A. schaalii* is a pathogen of the urinary tract and can cause systemic infections, such as CAUTI, urosepsis, pyelonephritis, and osteomyelitis [2,5,33,34]. Considering that *A. schaalii* detection in many clinical cases is associated with additional pathogens, *A. schaalii* has been termed a UTI co-pathogen [35]. We noticed that a comparison of database searches with *A. schaalii* versus *A. massiliense* of the same dataset resulted in 50-fold less protein identifications for *A. schaalii*, suggesting that these bacteria are phylogenetically more different than currently thought. A deeper genomic characterization should follow to determine classification of *A. massiliense* as a species of the newly introduced genus *Actinotignum* [6]. *A. massiliense* may share the co-pathogen definition as a cause of UTI and residence in CAUTI biofilms similar to the role ascribed to *A. schaalii* [36]. But it may also have a microbial bystander role in medical device-adapted biofilms. Joint presence of *Actinotignum/Actinobaculum spp.* and *A. urinae* was reported in several infection case reports suggesting their cooperative behavior in the human host environment. Other bacteria, such as *E. coli*, *P. mirabilis*, and *E. faecalis*, were also co-identified in clinical cases [2,5,34]. Those data are consistent with our findings on pathogens cohabitating CBs of two long term-catheterized patients. Shotgun proteomics is a direct diagnostic technique for *Actinotignum/Actinobaculum spp.* present in clinical urine specimens.

Innate immune responses, with neutrophils as the major cell type producing cytokines and effector molecules, are responsible for the defense against pathogens invading the human urinary tract [37]. The complement system and fibrinogen are also factors in the pathogenesis of UTI and CAUTI [38,39]. Pyuria and neutrophil invasion occur in CAUTIs that are asymptomatic [14,40]. The patients we studied were asymptomatic. In the five surveyed proteomes, there was evidence for the infiltration of neutrophils, the release of complement factors and the deposition of fibrinogen on catheter surfaces. Our data did not allow us to assess if the host defensive processes were caused by all present bacteria or only a subset thereof. It is likely that the immune system targets the entire microbial community in catheter biofilms. The *A. massiliense* proteome, and the comparison of in vivo and in vitro data, suggest that this bacterium not only adapts to the nutritional milieu in CBs but also to the confrontation with hostile host effector proteins. In the CB environment, the two *A. massiliense* strains expressed high quantities of putative host-interacting molecules (the Rib/alpha/Esp surface antigen repeat-containing proteins 00826 and 00827/00828 with cadherin-like adhesion domains), the latter of which contained a LPXTG cell wall localization motif, and two other LPXTG motif proteins with no functional predictions (00866 and 00581). Many characterized LPXTG motif proteins are involved in adhesion, invasion, and biofilm formation in, to name a few pathogens, *Staphylococcus aureus* [41], *Enterococci* [30,42], and *Listeria monocytogenes* [43]. We hypothesize that *A. massiliense* expresses these proteins at the cell surface to adhere to other bacterial cells, the abiotic latex surface and/or host proteins that are either exposed on the urothelial surface or deposited as part of the biofilm matrix on catheter surfaces. Specific functional roles need to be identified in biochemical and cell biological follow-up experiments.

Of considerable interest is the high in vivo expression level of two subtilisin-like proteases which may cleave cytokines that create an inflammatory environment at the urethral mucosal interface or complement, neutrophil, and eosinophil effector proteins. An ortholog expressed by the gene *prtP* of *Lactobacillus paracasei* was characterized as a bacterial surface protein with proteolytic activities against interferon γ-induced protein 10 kDa (IP-10) and other cytokines [25]. IP-10 is a key cytokine in infectious disease pathogenesis [44]. The protease PrtB was brought into context with a potential anti-inflammatory activity in irritable bowel disease (IBD) by degrading inflammatory cytokines. We hypothesize that lactocepin-like *A. massiliense* proteases (01095 and 00810) have proteolytic activities that modulate inflammation and enhance the microbe’s ability to persist as a biofilm constituent in CBs. The bacterium expresses a predicted PKS that may synthesize a molecule with polyketide [45] or mixed polyketide-peptide [46] structure. Such molecules have diverse antimicrobial, antitumor or anti-inflammatory bioactivities [47]. Stimulating research suggests that siderophores with a similar biosynthetic origin and known to capture bivalent metal ions in the extracellular milieu, are involved in proinflammatory signaling and possess virulence properties [48]. The *E. coli* proteomes in CBs of P1 and P5 showed evidence of the expression of enzyme components for the synthesis of two known siderophores, enterobactin and yersiniabactin.

Finally, the proteomic data allowed us to predict the *A. massiliense* metabolism and energy generation in the CB milieu. Our data strongly supported a preference of anaerobic energy generation pathways because many MAF enzymes were abundant while TCA cycle enzymes and cytochromes were absent. Among the sugars that are fed into glycolytic pathways, there was evidence for the use of pathways for the import and metabolism of xylose, glucoronate and glucarate. In non-diabetic patients, glucose is present in low concentrations in urine. In contrast, xylose and glucoronate are sugars present in glycoproteins and proteoglycans of the urethral, bladder, and renal epithelia [49]. Glucuronate conjugates are produced in kidneys where enzymes covalently couple the organic acid to lipophilic toxins to facilitate excretion with the urine. Oligopeptides are other kidney excretion products not exhaustively reabsorbed by the tubular system. We identified Mpp-type oligopeptide transport systems and peptide-degrading proteases at high expression levels in CBs. In summary, *A. massiliense* appears to adjust its metabolism to the nutrient repertoire available in urine and the urothelial mucosa. Studies of *E. faecalis* and *E. coli* grown in urine in vitro also reported high expression levels of peptide uptake systems [13,50]. The fitness of *E. coli* as a pathogen of the urinary tract was linked to its ability to take up and metabolize glucuronates and sugars present in urothelial glycoconjugates that are fed into the central carbon metabolism [11]. *A. massiliense* expressed enzymes contributing to glycogen synthesis, suggesting transient storage of this carbohydrate until this energy reservoir needs to be mobilized. In summary, our data support the notion that *A. massiliense* is well-adapted to the nutritional milieu in the urinary tract. It is plausible that the bacterium acquires and provides nutrients from microbial cohabitants in the biofilms. Further studies are needed to verify how CB cohabitants share metabolites and create a mutualistic, perhaps cooperative environment.

## Figures and Tables

**Table 1:** *Actinobaculum massiliense* proteins potentially participating in the crosstalk with the host.

Gene Locus ^1^	Protein Description ^2^	Functional Group or Domain ^3^	Put. Role in Inter-Action with Host ^4^	Predict. Location ^5^	Q (ivv vs ivt) ^6^	Q Avg (ivv) ^7^
01095	Putative subtilisin-like protease	fibronectin type III, S8pro	invasion, inflammation	CW; SP motif	>8	0.0617
00826	Rib/alpha/Esp surface antigen repeat-containing protein	Ca^2+^/cadherin bndg type III repeat and Ig-like fold	adhesion, biofilm formation	CW; SP motif	>8	0.0434
00810	Putative subtilisin-like protease	fibronectin type III, S8pro	invasion and inflammation	CW; SP motif	2–8	0.0406
01185	Oligopeptide/nickel binding protein	ABC transporter su., MppA–type	metal/heme/peptide uptake	CW; SP motif	2–8	0.0230
01650	l–arginine:glycine amidinotransferase	creatine synthesis from arginine	part of PKS pathway	CY	>8	0.0196
00827	Rib/alpha/Esp surface antigen repeat-containing protein	Ca^2+^/cadherin bndg type III repeat	adhesion, biofilm formation	CW; SP motif	2–8	0.0092
01410	Bacterial Ig-like domain protein	Ig-like domain	adhesion	not predicted	2–8	0.0047
01413	Listeria-Bacteroides repeat domain	Cadherin E-binding domain	adhesion, invasion	CW; SP motif	2–8	0.0045
01648	Ornithine carbamoyltransferase (ArcB)	arginine metabolism	part of PKS pathway	CY	>8	0.0042
00680	Oligopeptide/nickel binding protein	ABC transporter su., MppA-type	metal/heme/peptide uptake	CW; SP motif	<2	0.0038
01649	Carbamate kinase (ArcC)	arginine metabolism	part of PKS pathway	CY	>8	0.0034
01184	Oligopeptide ABC transporter, ATP-binding domain	ABC transporter su.	metal/heme/peptide uptake	CM	<2	0.0031
00954	Papain-like cysteine protease	cysteine protease	extracellular proteolysis	not predicted	2–8	0.0023
00866	LPXTG-domain-containing cell wall anchor protein	pilin subunit D1 domain	adhesion	LPLTG CW anchor	2–8	0.0022
01647	Arginine deiminase (ArcA)	arginine metabolism	part of PKS pathway	CY	>8	0.0021
01182	Oligopeptide ABC transporter, permease	ABC transporter su.	metal/heme/peptide uptake	CM	2–8	0.0017
00677	Oligopeptide ABC transporter, ATP-binding domain	ABC transporter su.	metal/heme/peptide uptake	CM	<2	0.0017
01364	Putative polyketide synthase	multifunctional enzyme	polyketide biosynthesis	CY	<2	0.0015
00649	Fe/B12 periplasmic binding protein	ABC transporter su., FecB-like	metal/cofactor uptake	CW; SP motif	>8	0.0015
00581	LPXTG-domain-containing cell wall anchor protein	G5 repeat domains	cell surface modulation	LPHTG CW anchor	>8	0.0014
01361	Biotin-[acetyl-CoA-carboxylase] ligase	part of PKS pathway	part of PKS pathway	CY	<2	0.0012
00678	Oligopeptide ABC transporter, ATP-binding domain	ABC transporter su.	metal/heme/peptide uptake	CM	<2	0.0010
01183	Oligopeptide ABC transporter, permease	ABC transporter su.	metal/heme/peptide uptake	CM	<2	0.0005
01418	Oligopeptide/nickel binding protein	ABC transporter su., MppA-type	metal/heme/peptide uptake	CW; SP motif	>8	0.0004
00679	Oligopeptide ABC transporter, permease	ABC transporter su.	metal/heme/peptide uptake	CM	<2	0.0004
01362	ATP grasp family protein	part of PKS pathway	part of PKS pathway	CY	<2	0.0003

Proteins are listed according to abundance in vivo. ^1^ gene locus (prefix HMPREF9233_, ^2^ descriptions from the protein annotation or that of an orthologous protein; ^3^ functional role assignments are based on the entire sequence or a domain (data were from UniProt, GO terms and InterPro references), su. = subunit; ^4^ putative interactions with the host based on data from ^2, 3, 5^, PKS = polyketide synthesis; ^5^ predicted subcellular localizations based on signal sequences for export or (LPXTG) cell wall anchor motifs, CY = cytosol, CW = cell wall, CM = cell membrane, SP = signal peptide; ^6^ range of abundance ratio in vivo (ivv) vs. in vitro (ivt); ^7^ estimated relative protein quantity averaged from four in vivo (ivv) datasets using the quotient PSMi/∑PSM.

**Table 2:** Abundant human proteins in catheter biofilm extracts with *A. massiliense* contributions.

Accession ^1^	Description ^2^	Average CB ^3^
P02768	^6^ Serum albumin = ALB [ALBU_HUMAN]	0.0793
P02788	^4,7^ Lactotransferrin = LTF [TRFL_HUMAN]	0.0365
P13645	^5^ Keratin, type I cytoskeletal 10 = KRT10 [K1C10_HUMAN]	0.0333
P05164	^4^ Myeloperoxidase = MPO [PERM_HUMAN]	0.0314
P06702	^4^ Protein S100-A9 = S100A9 [S10A9_HUMAN]	0.0306
P04264	^5^ Keratin, type II cytoskeletal 1 = KRT [K2C1_HUMAN]	0.0293
P01834	^6,8^ Immunoglobulin kappa constant chain = IGKC [IGKC_HUMAN]	0.0200
P0DOX5	^6,8^ Immunoglobulin gamma-1 heavy chain [IGG1_HUMAN]	0.0191
P02538	^5^ Keratin, type II cytoskeletal 6A = KRT6A [K2C6A_HUMAN]	0.0187
P04259	^5^ Keratin, type II cytoskeletal 6B = KRT6B [K2C6B_HUMAN]	0.0173
P35908	^5^ Keratin, type II cytoskeletal 2 epidermal = KRT2 [K22E_HUMAN]	0.0167
P59665	^4,7^ Neutrophil defensin = DEFA1 [DEF1_HUMAN]	0.0165
P02787	^6^ Serotransferrin = TF [TRFE_HUMAN]	0.0151
P0DOX7	^6,8^ Immunoglobulin kappa light chain [IGK_HUMAN]	0.0151
P01024	^8^ Complement C3 =C3 [CO3_HUMAN]	0.0146
P13646	^5^ Keratin, type I cytoskeletal 13 = KRT13 [K1C13_HUMAN]	0.0143
P13647	^5^ Keratin, type II cytoskeletal 5 OS = KRT5 [K2C5_HUMAN]	0.0137
P02533	^5^ Keratin, type I cytoskeletal 14 = KRT14 [K1C14_HUMAN]	0.0121
P08779	^5^ Keratin, type I cytoskeletal 16 = KRT16 [K1C16_HUMAN]	0.0116
P01876	^6,8^ Immunoglobulin heavy constant alpha 1 = IGHA1 [IGHA1_HUMAN]	0.0114
P02675	^8^ Fibrinogen beta chain = FGB [FIBB_HUMAN]	0.0112
P02679	^8^ Fibrinogen gamma chain = FGG [FIBG_HUMAN]	0.0109
P01861	^6,8^ Immunoglobulin heavy constant gamma 4 = IGHG4 [IGHG4_HUMAN]	0.0107
P05109	^4^ Protein S100-A8 = S100A8 [S10A8_HUMAN]	0.0101
P08311	^4^ Cathepsin G = CTSG [CATG_HUMAN]	0.0101

Proteins are ordered based on average abundance in four in vivo CB proteomic datasets with evidence of *A. massiliense* colonization. ^1^ UniProt ID; ^2^ descriptions from annotation; ^3^ normalized quantities derived from four CB datasets using the PSMi/∑PSM quotient; ^4^ proteins abundant in activated neutrophils; ^5^ proteins abundant in keratinizing epithelial cells and variably expressed by urothelial cells; ^6^ proteins abundant in normal urine; ^7^ proteins secreted by urothelial cells upon pathogen recognition; ^8^ proteins released during the inflammatory response in injured tissues. The information on cell-specific expression in the urinary tract, extracellular release and presence in normal urine (after glomerular filtration) is derived from Protein Atlas (www.proteinatlas.org) and literature [27,28,29].

## Supplementary Materials

The supplementary materials are available online at http://www.mdpi.com/2227-7382/6/4/52/s1.
