# Supplementary material for: Actinobaculum massiliense Proteome Profiled in Polymicrobial Urethral Catheter Biofilms
Source: Proteomes. 2018 Dec 9;6(4):52. doi: 10.3390/proteomes6040052 (PMC6314084; doi:10.3390/proteomes6040052)
Supplement: Supplementary file 1 [file proteomes-06-00052-s001.zip › Suppl Materials File S1_rev.pdf]

Suppl. Materials, Table S1 (File S1). Metaproteomic database searches and entire genome sequence-derived protein sequence entries (ORFs) from Homo Sapiens and microbial species colonizing the human urogenital tract, bladder catheters and cause urinary tract infections.

No.	Microbial species and strain	UniProt web link	Number of ORFs / proteins	Taxon identifier
1	Homo sapiens (Reviewed)	http://www.uniprot.org/taxonomy/9606	20,259	9606
2	Actinotignum schaalii FB123-CNA-2	http://www.uniprot.org/taxonomy/883067	1,726	883067
3	Aerococcus urinae (strain ACS-120-V-Col10a)	http://www.uniprot.org/taxonomy/866775	1,684	866775
4	Bacteroides fragilis (strain 638R)	http://www.uniprot.org/taxonomy/862962	4,284	862962
5	Candida albicans (strain WO-1)	http://www.uniprot.org/taxonomy/294748	5,742	294748
6	Citrobacter koseri (strain ATCC BAA-895 / CDC 4225-83 / SGSC4696)	http://www.uniprot.org/taxonomy/290338	5,020	290338
7	Corynebacterium urealyticum (strain ATCC 43042 / DSM 7109)	http://www.uniprot.org/taxonomy/504474	2,011	504474
8	Enterobacter cloacae subsp. cloacae (strain ATCC 13047 / DSM 30054 / NBRC 13535)	http://www.uniprot.org/taxonomy/716541	5,411	716541
9	Enterococcus faecalis (strain ATCC 700802 / V583)	http://www.uniprot.org/taxonomy/226185	3,240	226185
10	Escherichia coli (strain UTI89 / UPEC)	http://www.uniprot.org/taxonomy/364106	5,192	364106
11	Gardnerella vaginalis (strain ATCC 14019 / 317)	http://www.uniprot.org/taxonomy/525284	1,365	525284
12	Klebsiella pneumoniae (strain 342)	http://www.uniprot.org/taxonomy/507522	5,738	507522
13	Lactobacillus gasseri (strain ATCC 33323 / DSM 20243)	http://www.uniprot.org/taxonomy/324831	1,694	324831
14	Morganella morganii subsp. morganii KT	http://www.uniprot.org/taxonomy/1124991	3,510	1124991
15	Prevotella melaninogenica D18	http://www.uniprot.org/taxonomy/575612	2,461	575612
16	Proteus mirabilis (strain HI4320)	http://www.uniprot.org/taxonomy/529507	3,661	529507
17	Providencia stuartii (strain MRSN 2154)	http://www.uniprot.org/taxonomy/1157951	4,219	1157951
18	Pseudomonas aeruginosa (strain PA7)	http://www.uniprot.org/taxonomy/381754	6,246	381754
19	Serratia marcescens WW4	http://www.uniprot.org/taxonomy/435998	4,801	435998

20	Staphylococcus aureus (strain USA300 / TCH1516)	http://www.uniprot.org/taxonomy/451516	2,694	451516
21	Staphylococcus saprophyticus subsp. saprophyticus (strain ATCC 15305 / DSM 20229)	http://www.uniprot.org/taxonomy/342451	2,404	342451
22	Streptococcus agalactiae serotype V (strain ATCC BAA-611 / 2603 V/R)	http://www.uniprot.org/taxonomy/208435	2,105	208435
23	Mycoplasma genitalium (strain ATCC 33530 / G-37 / NCTC 10195)	http://www.uniprot.org/taxonomy/243273	484	243273
24	Ureaplasma urealyticum serovar 10 (strain ATCC 33699 / Western)	http://www.uniprot.org/taxonomy/565575	646	565575
25	Staphylococcus saprophyticus subsp. saprophyticus (strain ATCC 15305 / DSM 20229)	http://www.uniprot.org/taxonomy/342451	2,404	342451
26	Actinobaculum massiliense ACS-171-V-Col2	http://www.uniprot.org/taxonomy/883066	1,696	883066
27	Propionimicrobium lymphophilum ACS-093-V-SCH5	http://www.uniprot.org/taxonomy/ 883161	2,076	883161

Microbial protein sequence databases are listed with their UniProt proteome names, links and numerical taxon identifiers. The database searches identified experimentally generated tryptic peptide fragments via peptide spectral matches with tryptic peptides *in silico* processed from the protein sequences annotated in the UniProt proteomes. In the initial stages, searches were performed with this common metaproteomic database until selective searches were introduced to reflect the true presence of all moderate to high abundance microbes in a clinical sample. Thus, the searches are computational comparisons of LC-MS/MS spectra with predicted mass spectral patterns. *Actinobaculum massiliense*, added for the purpose on the specific interest in its proteome for this publication, is listed as the final species/strain and associated database.
