# Supplementary figures and images for: Actinobaculum massiliense Proteome Profiled in Polymicrobial Urethral Catheter Biofilms
Source: Proteomes. 2018 Dec 9;6(4):52. doi: 10.3390/proteomes6040052 (PMC6314084; doi:10.3390/proteomes6040052)

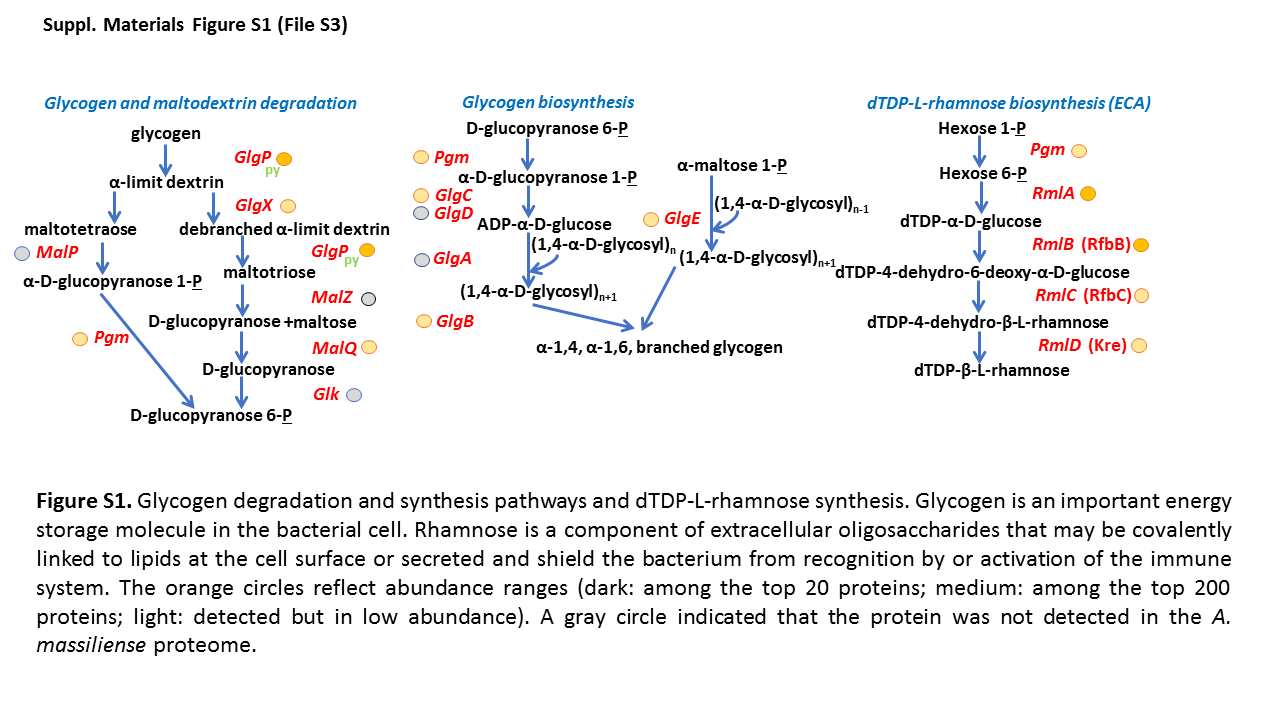

Supplement: Supplementary file 1 [file proteomes-06-00052-s001.zip › Suppl Materials File S3.tif]
